# Clozapine-Induced Myocarditis or Acute Coronary Syndrome? Optical Coherence Tomography to the Rescue

**DOI:** 10.1155/2018/5026107

**Published:** 2018-07-19

**Authors:** Ayodeji Dina, Peter Barlis, William van Gaal

**Affiliations:** ^1^Department of Cardiology, Northern Hospital, 185 Cooper Street, Epping, VIC 3076, Australia; ^2^Faculty of Medicine, Dentistry and Health Sciences, Melbourne Medical School, The University of Melbourne, 175 Cooper Street, Epping, VIC 3076, Australia

## Abstract

Chest pain and troponin elevation may be due to an acute coronary syndrome, myocarditis, acute cardiomyopathy, or other less common conditions. Management differs depending on the aetiology, and the pathophysiologic diagnosis has direct implications on treatment and patient outcomes. History and clinical examination is supplemented by selected investigations including the electrocardiogram, chest X-ray, echocardiography, coronary angiography, and even myocardial perfusion scintigraphy or cardiac magnetic resonance imaging. Intravascular imaging can provide important insights into the underlying mechanism of acute coronary syndromes, especially when angiography is ambiguous.

## 1. Introduction

We report a case of chest pain initially thought to be clozapine-induced myocarditis that remained undiagnosed after multiple imaging modalities were performed. Only optical coherence tomography (OCT) was able to provide the correct diagnosis and enable appropriate management.

## 2. Presentation

A 58-year-old female with hypertension, hyperlipidaemia, and type two diabetes presented with atypical central chest pain seven days after commencing clozapine for refractory schizophrenia. She had trialled multiple antipsychotic medications as well as electroconvulsive therapy without success; however her symptoms did improve after commencing clozapine. On physical examination, her vital signs were normal with a heart rate of 90, regular, and blood pressure of 110/70. Her cardiovascular examination was normal. There were no murmurs and no pericardial rub. Her ECG on admission was normal. Her troponin levels increased from <0.01 *μ*g/L before commencement of clozapine therapy to 0.13 *μ*g/L on admission, peaked to 0.14 *μ*g/L, and fell to <0.01 *μ*g/L on discharge (normal range < 0.04 *μ*g/L). Other laboratory investigations were within normal limits. Her transthoracic echocardiogram revealed normal LV size and systolic function with no regional wall motion abnormality. Clozapine-induced myocarditis was suspected; however, a cardiac magnetic resonance scan showed no late gadolinium enhancement or increased signal intensity on T1- and T2-weighted images to suggest myocarditis. A stress myocardial perfusion scan revealed a small area of reversible ischemia in the distal lateral wall only.

**Figure 1:**
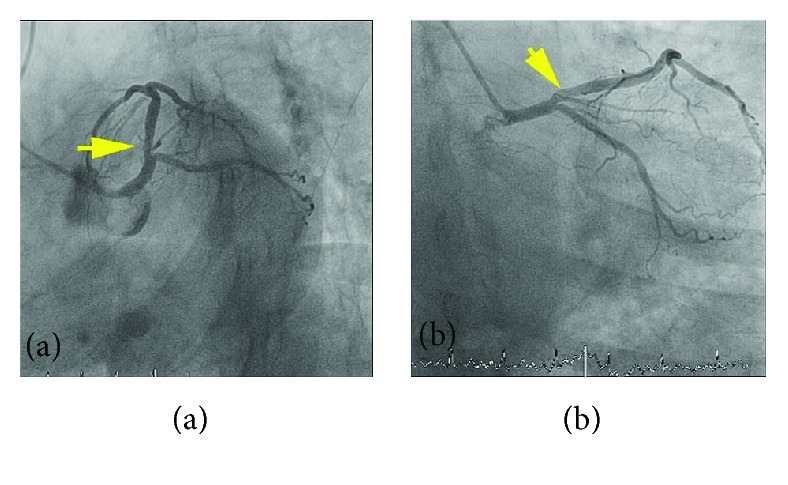
Angiographic view of the left anterior descending artery. (a, b) Mildly hazy lesion in the ostial LAD (yellow arrow).

**Figure 2:**
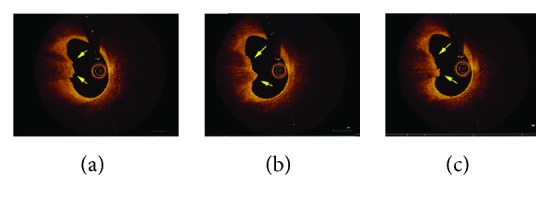
Representative OCT images of the culprit lesion. (a, b, c) Plaque erosion: adjacent thrombus (yellow arrows) overlying an intact fibrous cap.

Coronary angiography was performed which revealed a mildly hazy lesion in the ostial left anterior descending artery with normal TIMI-3 flow (Figures 1(a) and 1(b)). To further characterize this lesion, OCT was performed demonstrating a region of lipid-rich plaque, intact fibrous cap with thickness of 100 *μ*m, and adjacent thrombus consistent with plaque erosion (Figures 2(a)–2(c)). The minimal lumen area at the ostial LAD was 5.24 mm^2^. Given that the lesion was not flow limiting and she was asymptomatic, we decided to manage her medically.

The patient was commenced on dual antiplatelet therapy and her clozapine which had been stopped on admission was restarted. We followed her up clinically in the outpatient department, and she remained asymptomatic during her 3-month follow-up.

## 3. Discussion

Most patients that present to the hospital with chest pain and elevated troponin are diagnosed with myocardial infarction. About 6% of these patients do not have significant coronary lesions on angiography [1] making it difficult to establish a precise diagnosis. Establishing a precise diagnosis has both therapeutic and prognostic implications for patients. In 95% of cases with normal coronary arteries, the aetiology includes acute myocarditis, acute myocardial infarction, and cardiomyopathy especially Takotsubo [2, 3]. Similarity in the clinical presentation of acute coronary syndromes and myocarditis contributes to the diagnostic and management dilemma of these patients.

Myocarditis is the commonest underlying aetiology in patients with suspected acute coronary syndrome with nonobstructive coronary arteries and accounts for up to three quarters of cases in several studies [2, 3]. Although the incidence of clozapine-induced myocarditis is about 0.7–1.2% in a previous study [4], there should be a high index of suspicion, especially in our case given the temporal association of commencing clozapine to the patient's symptoms. In cases of suspected clozapine-induced myocarditis, it is recommended that clozapine is not rechallenged [5]. However, clozapine is recommended for refractory schizophrenia; therefore, stopping it may pose a therapeutic challenge.

Echocardiography, myocardial perfusion scintigraphy, and cardiac magnetic resonance imaging are valuable tools in establishing a diagnosis in ambiguous cases. Echocardiographic findings of acute coronary syndromes include regional wall motion abnormalities and left ventricular dysfunction; however, this may also be present in myocarditis. As such, echocardiography lacks specificity. Myocardial perfusion scintigraphy is important in assessing the presence, location, and severity of myocardial ischemia. The small area of reversible ischemia in the distal lateral wall in our case may represent a false-positive study or distal embolization of thrombus with microvascular plugging.

CMR, especially, has increasingly been used in establishing a diagnosis in these conditions, as it is able to detect areas of myocardial infarction and visualise fibrotic and necrotic areas. However, the diagnostic accuracy is variable. In a study by Pathik et al., cardiac MR was unable to establish a diagnosis in 13% of patients with troponin-positive chest pain and nonobstructive coronary arteries [6]. In a case series of patients with suspected clozapine-induced myocarditis who presented with a normal ECG and echocardiogram, cardiac MR was unremarkable [7].

Patients with troponin-positive chest pain often undergo coronary angiography during admission. Nonobstructive coronary artery disease on angiography makes a vascular cause of infarction less likely and, however, does not exclude it completely as evidenced in our case. The underlying aetiology in up to 40% of patients presenting with troponin-positive chest pain and nonobstructive coronary arteries is plaque disruption [8, 9]. Plaque rupture and plaque erosion are the most common pathophysiological mechanisms of acute coronary syndrome. Intravascular imaging techniques like intravascular ultrasound (IVUS) and optical coherence tomography have greatly improved our ability to characterize underlying mechanism of ACS. While IVUS is the most common imaging technique used in clinical practice, it has 10 times lower resolution compared to OCT and is therefore unable to accurately visualise superficial intravascular dimensions. Hence, OCT is currently the only imaging modality available to identify plaque erosion in vivo. It uses a light source with a wavelength between 1280 and 1350 nm measuring backscattered light producing cross-sectional images of arteries [10]. OCT produces near in vivo histological images of the superficial layers of the coronary vessel wall and is considered to be more accurate in characterizing the fibrous cap and thrombosis when compared to other intravascular imaging techniques [10].

Plaque rupture is characterized by intraluminal thrombus on a disrupted thin fibrous cap overlying a necrotic core with infiltration of macrophages whereas plaque erosion manifests as thrombus on an intact fibrous cap with less or deep-seated necrotic core. Apart from this morphological difference, Ferrante et al. also reported biomarker difference between plaque rupture and plaque erosion [11]. Their study showed a higher level of serum myeloperoxidase in patients with plaque erosion when compared to plaque rupture [11]. This differentiation may have clinical outcome implications as patients with plaque erosion may have lower rates of disease progression and revascularization [12].

While management of ACS has relied mainly on catheter-based reperfusion and stenting of the infarct-related artery, OCT has now enabled us to identify the difference in underlying plaque morphology between plaque rupture and erosion. Given that stenosis in plaque erosion is not always significant and there is no disruption to the vessel wall, medical management with dual antiplatelet therapy only may be an alternative to coronary stenting in patients without significant stenosis, avoiding potential early and late stent complications. This is supported by a prospective study by Jia et al. which showed >50% reduction in thrombus volume at 1 month in ACS patient with plaque erosion managed with antithrombotic therapy only [13]. In addition, in a retrospective study of 31 STEMI patients, none of the 12 patients with plaque erosion managed with thrombectomy and dual antiplatelet therapy only required further revascularization in the 2-year follow-up [14]. Our patient was managed medically and has remained asymptomatic at clinic follow-up.

## 4. Conclusion

This case underscores the importance of OCT in clarifying the diagnosis in patients with acute coronary syndromes and angiographically nonobstructive coronary artery disease. In our case, OCT outperformed cardiac MRI, perfusion imaging, and angiography. OCT was the only modality to provide a pathophysiologic diagnosis and the only investigation to have direct treatment implications, allowing for clozapine therapy to continue and evidence-based ACS treatment to be commenced.

Also, the incidence of coronary plaque disruption may be underestimated in clozapine-treated patients who present with troponin-positive chest pain. Further investigations including coronary angiography and OCT may be important in selected patients with suspected clozapine-induced myocarditis, who have a nondiagnostic CMR.
